# Regulation of Clusterin in the Heart and Plasma of Mice After Transverse Aortic Constriction

**DOI:** 10.1111/jcmm.70290

**Published:** 2024-12-13

**Authors:** Annie Turkieh, Lukas Weber, Maggy Chwastyniak, Simge Baydar, Olivia Beseme, Matthias Ernst, Quianling Ye, Philippe Amouyel, Bruno K. Podesser, Attila Kiss, Florence Pinet

**Affiliations:** ^1^ Inserm, CHU Lille, Institut Pasteur de Lille, U1167‐RID‐AGE Université de Lille Lille France; ^2^ Ludwig Boltzmann Institute for Cardiovascular Research at the Center for Biomedical Research and Translational Surgery Medical University of Vienna Vienna Austria

**Keywords:** cardiac remodelling, clusterin, heart failure, pressure overload, TAC mice

## Abstract

Chronic pressure overload induces adverse cardiac remodelling characterised by left ventricular (LV) hypertrophy and fibrosis, leading to heart failure (HF). Identification of new biomarkers for adverse cardiac remodelling enables us to better understand this process and, consequently, to prevent HF. We recently identified clusterin (CLU) as a biomarker of cardiac remodelling and HF after myocardial infarction. The aim of this study was to investigate whether CLU expression is regulated in the heart and could be used as an indicator of adverse cardiac remodelling in response to pressure overload. We quantified CLU in the LV of mice subjected to transverse aortic constriction (TAC) and observed increased CLU mRNA levels and its mature protein form (m‐CLU) compared to the sham. Interestingly, CLU mRNA levels were positively correlated with pro‐hypertrophic (ANP, BNP, B‐MHC), pro‐ and anti‐fibrotic (*TGFb*, *ColI* and *CILP*) genes. In addition, m‐CLU was positively correlated with LV hypertrophy, LV end diastolic and systolic diameters, and negatively correlated with LV ejection fraction. Finally, we observed that m‐CLU levels only increased in TAC mice with severe cardiac remodelling and dysfunction without any significant difference in plasma CLU levels. This is the first study to demonstrate that cardiac expression of CLU is induced in the LV of TAC mice during adverse cardiac remodelling. However, plasma CLU levels could not be used as biomarkers of TAC‐induced cardiac remodelling and dysfunction.

## 1 Background

Cardiac remodelling is defined as genomic expression resulting in molecular, cellular and interstitial changes that manifest clinically as changes in cardiac size, shape, and volume [1, 2]. It can occur in response to pressure overload, such as aortic stenosis and hypertension, or injury, such as myocardial infarction (MI), to maintain cardiac function, but in the long term contributes to the development of heart failure (HF) [3, 4, 5]. Despite the therapeutic advances, HF is still one of the leading causes of mortality worldwide and remains a major clinical and public health problem [6, 7]. The identification of new biomarkers for adverse cardiac remodelling will allow us to better understand this process and thereby contribute to the prevention of HF.

Clusterin (CLU) is a secreted glycoprotein that is constitutively expressed in most mammalian tissues and is highly conserved across species [8]. CLU has been shown to be induced in the heart during cardiac injury (MI [9, 10, 11] and myocarditis [12]) and appears to play a protective role by reducing cardiac inflammation and apoptosis [13, 14]. Recently, we identified CLU as a biomarker for cardiac remodelling after MI and a predictor of early death in HF patients [15]. Indeed, by using a proteomic approach without a priori (2D‐DIGE electrophoresis) on plasma obtained from patients after a first anterior MI, we identified CLU to be increased in plasma of patients who developed a higher grade of adverse cardiac remodelling one year after MI compared to patients who had moderate adverse post‐MI cardiac remodelling. In addition, we quantified the circulating levels of CLU in patients with chronic HF using quantitative mass spectrometry and ELISA and observed an increase in its levels in the plasma of HF patients who died of cardiovascular causes during a 3‐year follow‐up compared with survivors [16]. CLU expression is also increased in the left ventricle (LV) of ischaemic HF patients and rats with MI and is positively correlated with pro‐hypertrophic genes and cardiac remodelling parameters such as LV weight and diameter. Furthermore, CLU expression and secretion are induced in isoproterenol‐treated cardiomyocytes and seem to play a prohypertrophic role by regulating the ERK signalling pathway [15]. These data suggest that CLU is a potential novel protein involved in cardiac remodelling and HF after cardiac insults, and its expression predicts the risk of HF development.

The aim of this study was to investigate whether CLU could be a biomarker for cardiac remodelling and dysfunction in response to pressure overload. To this end, CLU expression was quantified in the LV and plasma of mice subjected to transverse aortic constriction (TAC) for 6–8 weeks and correlated with echocardiographic parameters as well as with the expression of genes involved in hypertrophy and fibrosis, two major mechanisms observed during chronic pressure overload of the heart.

## 2 Materials and Methods

### 2.1 Animals

The experimental protocol was approved by the regional Ethics Committee for Laboratory Animal Experiments at the Medical University of Vienna and the Austrian Ministry of Science Research and Economy (BMWFW‐66.009/0205‐WF/V3b/2015), conforming with the Guide for the Care and Use of Laboratory Animals published by the US National Institutes of Health (NIH Publication No. 85–23, revised 1996).

### 2.2 Pressure Overload Induced Left Ventricular Hypertrophy Model

TAC surgery was performed in 10–12 week old male and female mice (C57Bl6/J, Charles River) for 6–8 weeks. Mice were anaesthetised by injection of medetomidine, midazolam, fentanyl and ketamine (MMFK). After intubation, mechanical ventilation was ensured with a respiratory rate of 150 per minute. Additionally, 1%–1.5% isoflurane was used to ensure sufficient anaesthesia depth throughout the whole procedure. The mice were placed in a supine position on a heating plate, and their extremities were fixed. To prevent dehydration of the eyes, ointment was applied. The surgery was performed under constant ECG surveillance as well as monitoring of respiratory rate and body temperature. The chest was opened via a median sternotomy starting cranially until the second rib. The thymus was dissected to provide access to the aortic arch. The ascending aorta was visualised and cleared from perivascular fat tissue, and then a 6‐0 silk suture was pulled under the ascending aorta and between the innominate artery and left common carotid artery. A spacer (27G needle) was placed below the suture to avoid complete occlusion of the aorta. The chest was closed using a 6‐0 Polysorb suture, and the skin was closed using a 4‐0 Polysorb suture. Postoperatively, MMFK was antagonised by s.c. injection of atipamezole and flumazenil. To support post‐op recovery, all animals received a single s.c. injection of 5% glucose solution. Mice were extubated when spontaneous respiration was detectable. For additional post‐op analgesia, mice received a single i.p. injection of buprenorphine. Additionally, mice received piritramide through the drinking water ad libitum for the first three days post OP.

Transthoracic echocardiography was performed using a Vevo 2100 and 3100 imaging system (Visualsonics) with a 55‐MHz transducer as described previously [17]. The mice were anaesthetised with 1%–1.5% isoflurane. Body temperature and ECG were continuously monitored throughout the measurement via limb electrodes and rectal probes, respectively. Parasternal long‐axis and short‐axis views were obtained, which were analysed to assess the LV dimension and function. The obtained ultrasound images and videos were analysed by Vevo LAB software, where a mean of 3 cardiac cycles in each view was used for each parameter.

### 2.3 RNA Extraction and Quantitative Real Time‐Polymerase Chain Reaction (qRT‐PCR)

RNAs were extracted from cardiac tissue with TRI Reagent (Ambion) as described by the manufacturer's instructions. RNAs were quantified using a NanoVue spectrophotometer and then retrotranscribed using the miScript II RT kit (QIAGEN). Indeed, 500 ng of RNA were mixed with 2 μL of reverse transcriptase enzyme, 2 μL of dNTP mix (10×), 4 μL of HiFlex buffer (5×) and sufficient DNAse/RNAse‐ free water for a total volume of 20 μL. Mixes were then incubated for 1 h at 37°C on the Biometra Gradient Thermal Cycler, followed by 5 min at 95°C. The cDNAs were then amplified with miScript SYBR Green PCR (QIAGEN) on an Aria Mx Q‐PCR system (Agilent Technologies). Indeed, 2.5 μL of diluted cDNA (1/20 or 1/40) was added to 12.5 μL of Sybergreen buffer, 2.5 μL of forward and reverse primers (10 μM) and 7.5 μL of RNAase/DNAase‐free water and amplified according to the following program: Step 1: 95°C/15 min, Step 2: 94°C/15 s, Step 3: primer melting temperature/30 s, and Step 4: 70°C/30 s. Steps 2–4 were repeated 40 times. The sequences and the melting temperature (MT) of the different primers (Eurogentec) used were detailed in Table S1. The ΔΔCT method was used for data analysis.

### 2.4 Protein Extraction and Western Blot Analysis (WB)

Protein extracts were collected by Dounce‐Potter homogenisation on ice‐cold RIPA buffer (50 mmol/L Tris pH 7.4, 150 mmol/L NaCl, 1% Igepal (Sigma, CA‐630), 50 mmol/L sodium deoxycholate, and 0.1% SDS) containing anti‐proteases (Roche, 11836145001/1 tablet for 50 mL of buffer), 1 mmol/L sodium orthovanadate (Sigma, S6508) and 1% protein phosphatase inhibitors cocktail 2 and 3 (Sigma, P5726 and P0044). All extracts were then incubated for one hour at 4°C and then centrifuged at 6,700 g, 4°C, for 10 min to remove cellular debris. Protein concentrations were determined with a Bradford‐based protein assay (Bio‐Rad, 500‐0006) according to the manufacturer's instructions. Ten μg of LV proteins and 1 μL of 1/10 diluted plasma were separated on 10% SDS‐PAGE and transferred on 0.22 μm nitrocellulose membranes (Trans‐Blot Turbo Transfert Pack, Bio‐Rad). The membranes were blocked with 5% non‐fat dry milk in TBS‐Tween 0.1% buffer for 1 h at room temperature and then were incubated with primary antibodies at 4°C with gentle shaking overnight in the blocking solution. The primary antibodies used were clusterin (1/2500 for LV and 1/20,000 for plasma; GeneTex, GTX88520) and GAPDH (1/20,000, GeneTex, GTX 100118). The membranes were then washed three times for 10 min with TBS‐Tween 0.1% buffer and incubated with the corresponding horseradish peroxidase‐labelled secondary antibody for 1 h in the blocking solution. The secondary antibodies used were anti‐goat IgG (1/5000 for LV and 1/40,000 for plasma; Santa Cruz Biotechnology, sc‐2354) and anti‐rabbit IgG (1/10,000; Jackson Immuno Research, 111‐035‐003). After three washes with TBS‐Tween 0.1% buffer, the membranes were incubated with enhanced chemiluminescence (ECL) western blotting detection reagents (Bio‐Rad) according to the manufacturer's instructions. The Chemidoc camera (Bio‐Rad) was then used for imaging the membranes, and densitometric measurements of the bands were analysed with the Image Lab software (Bio‐Rad).

### 2.5 Statistical Analysis

Data are expressed as individual values and means ± SEM and analysed with GraphPad software version 7.0 (GraphPad, San Diego, CA, USA). Comparisons were made by the Wilcoxon–Mann–Whitney test (2 groups) and by the Anova or Kruskal–Wallis test (4 groups, depending on the test of normality). Correlations were carried out by the Spearman correlation test. Results were considered statistically significant if the *p* < 0.05.

## 3 Results

### 3.1 CLU Expression Is Induced in Heart After TAC

**FIGURE 1:**
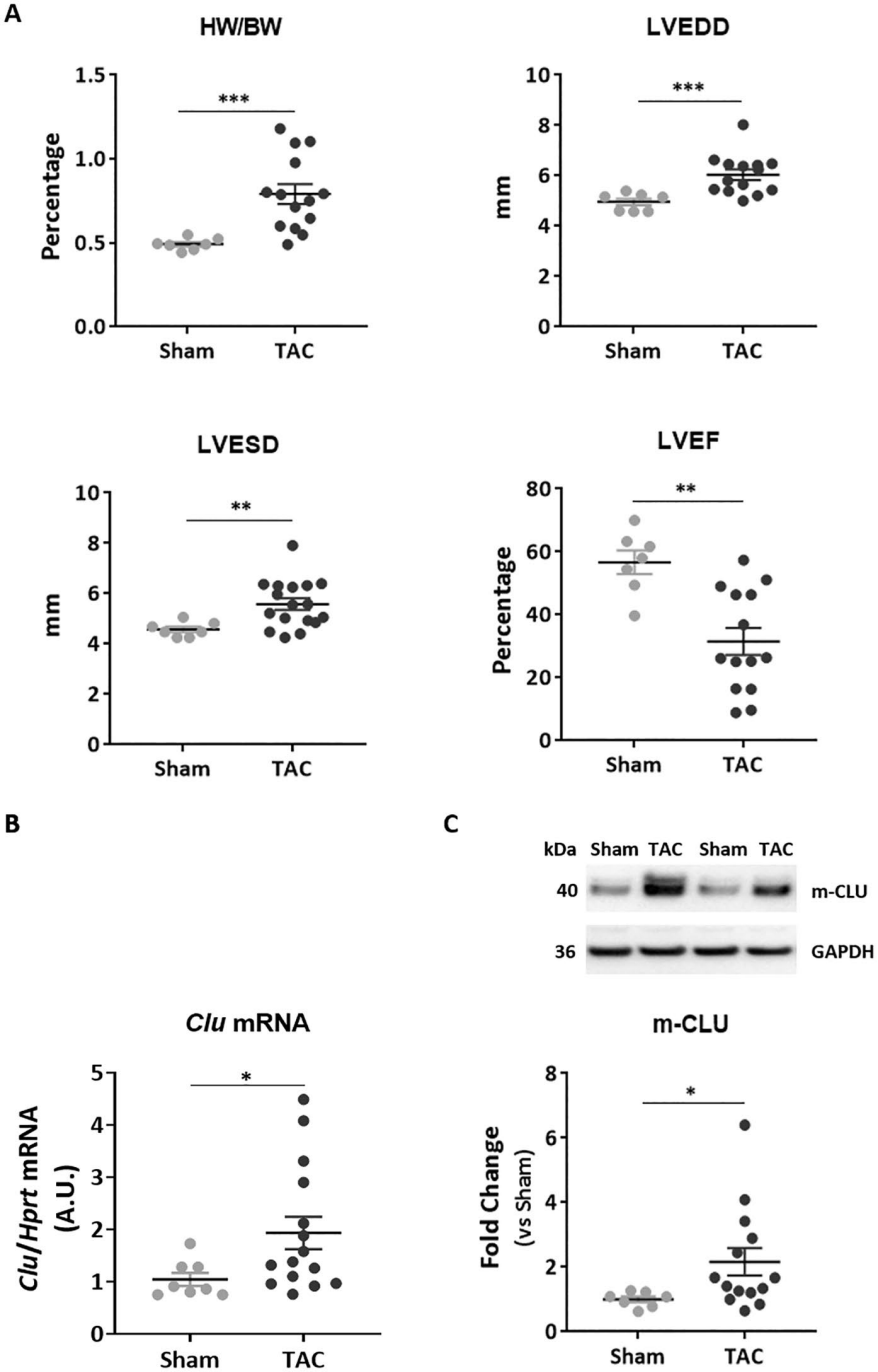
CLU expression is induced in the heart after transverse aortic constriction (TAC). (A) Animal characteristics after 6–8 weeks of TAC were quantified for heart weight/body weight (HW/BW) ratio, left ventricle end diastolic diameter (LVEDD), left ventricle end systolic diameter (LVESD), and ejection fraction (EF) in sham (*n* = 7) and TAC mice (*n* = 14). (B) Quantification of *Clu* mRNA level by qPCR in the left ventricle of sham and TAC mice. *Hprt* was used for normalisation. (C) Representative image and quantification of CLU mature protein form (m‐CLU) by western blot in the same samples. GAPDH was used for normalisation. Statistical significance was determined by the Wilcoxon‐Mann–Whitney test. *p < 0.05, **p < 0.01, and ***p < 0.001.

We used a TAC mouse model to study the effect of pressure overload on CLU expression in the heart. TAC for 6–8 weeks induced cardiac remodelling and dysfunction in most animals (78% and 64% of TAC animals, respectively), as observed by a significant increase in heart weight/body weight (HW/BW) ratio, left ventricular end‐diastolic (LVEDD) and systolic (LVESD) diameters, and a significant decrease in left ventricular ejection fraction (EF) in the TAC group compared with the sham group (Figure 1A). In this model, we demonstrated a significant increase in *Clu* mRNA (Figure 1B) levels and its mature protein form (m‐CLU) (Figure 1C) in the LV of TAC mice compared to sham mice.

### 3.2 Cardiac CLU Levels Are Positively Correlated to TAC Induced‐ Cardiac Hypertrophy

**FIGURE 2:**
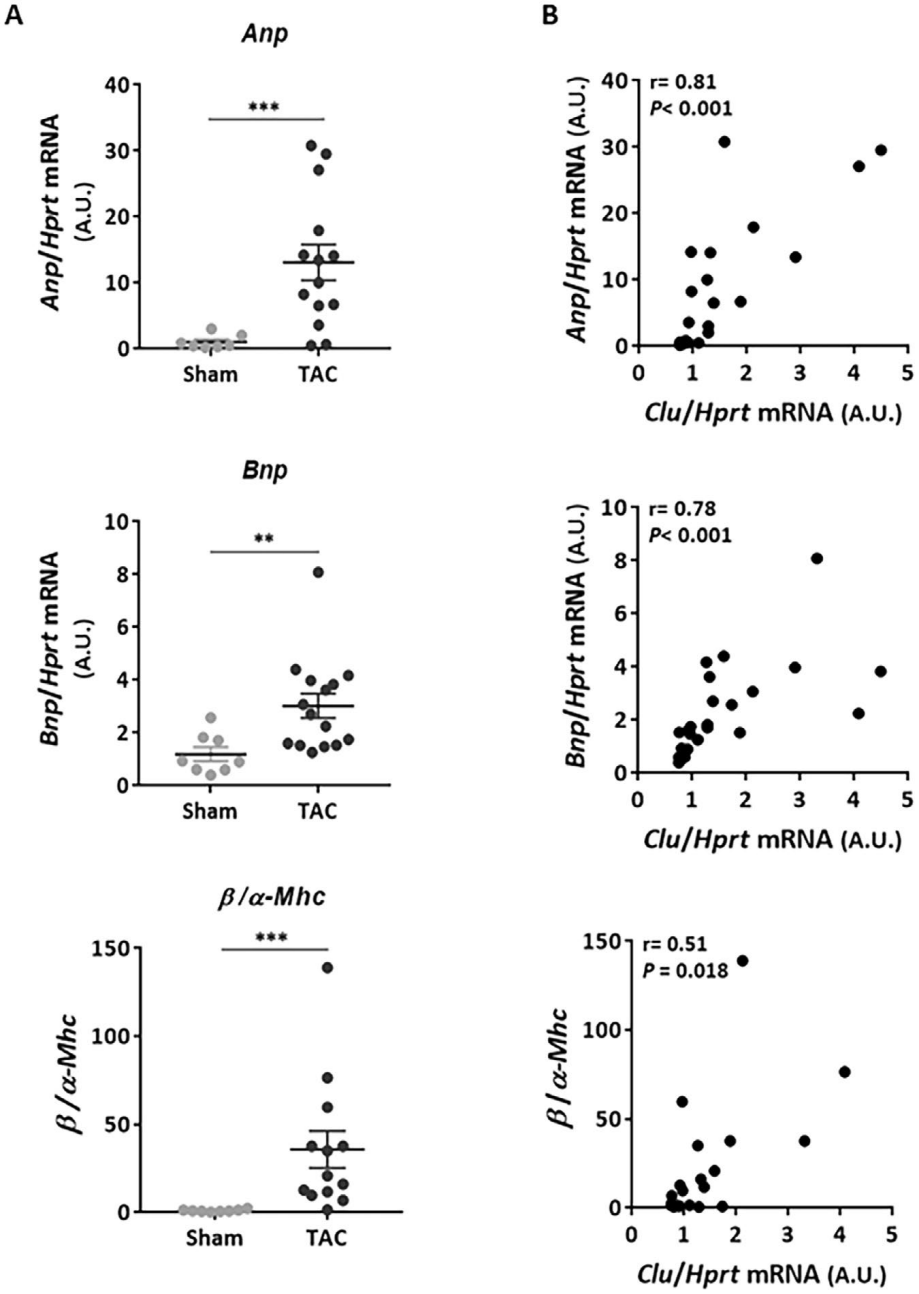
Cardiac CLU levels are positively correlated to cardiac hypertrophy induced by transverse aortic constriction. (A) Quantification by qPCR of mRNA levels of genes involved in hypertrophy in sham (*n* = 7) and TAC (*n* = 14) mice: *Anp* (atrial natriuretic peptide), *Bnp* (brain natriuretic peptide), and β/α‐*Mhc* (beta and alpha myosin heavy chain). *Hprt* was used for normalisation. Statistical significance was determined by the Wilcoxon‐Mann Whitney test. **p < 0.01, and ***p < 0.001. (B) Correlation between intraventricular *Clu* mRNA levels and those of *Anp*, *Bnp*, and β/α‐*Mhc*. Correlations were carried out by the Spearman correlation test. Results were considered statistically significant if the *p* < 0.05.

To better understand the relationship between CLU expression and cardiac remodelling and dysfunction, we first examined the correlation between intraventricular CLU expression and cardiac hypertrophy. Cardiac hypertrophy and stretch markers were also assessed by quantifying the expression of *Anp* (atrial natriuretic peptide), *Bnp* (brain natriuretic peptide), *α‐* and *β*‐*Mhc* (alpha and beta myosin heavy chain) by qPCR. In addition to the increase in HW/BW ratio (Figure 1A), we observed that *Anp*, *Bnp*, and *β*‐*Mhc* mRNA levels and the ratio *β*‐*Mhc*/*α*‐*Mhc* were significantly increased (Figure 2), but *α*‐*Mhc* mRNA levels were significantly decreased in the TAC mouse group compared to the sham group (Figure S1A). *Clu* mRNA levels were positively correlated with *Anp*, *Bnp*, and *β*‐*Mhc* mRNA levels (Figure 2B) without any correlation with *α*‐*Mhc* mRNA levels (Figure S1B). Furthermore, m‐CLU levels were positively correlated with the HW/BW ratio as a marker of LV hypertrophy (Figure 2B). These correlations were still significantly observed in TAC mice after exclusion of sham mice (Figure S1C). These results showed that CLU was positively correlated with cardiac hypertrophy and could be used as an indicator of excessive hypertrophy induced by pressure overload.

### 3.3 Cardiac CLU Levels Are Positively Correlated to TAC‐ Induced Cardiac Fibrosis

**FIGURE 3:**
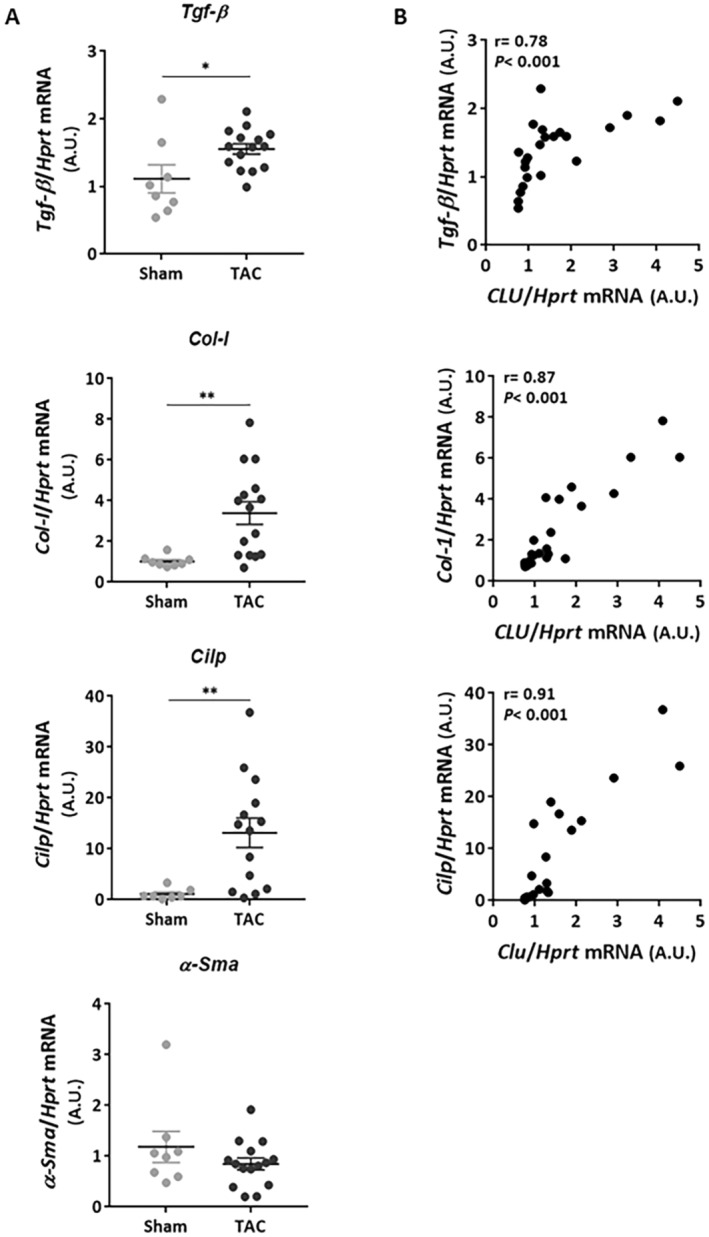
Cardiac CLU levels are positively correlated to cardiac fibrosis induced by transverse aortic constriction. (A) Quantification by qPCR of mRNA levels of genes involved in fibrosis regulation in sham (*n* = 7) and TAC (*n* = 14) mice: *Tgf‐β* (transforming growth factor‐beta), *Col I* (collagen type I), α‐*Sma* (alpha‐smooth muscle Actin); and the antifibrotic *Cilp* (cartilage intermediate layer protein‐1). *Hprt* was used for normalisation. Statistical significance was determined by the Wilcoxon‐Mann Whitney test. *p < 0.05, **p < 0.01. (B) Correlation between intraventricular *Clu* mRNA levels and those of *Tgf‐β*, *Col I* and *Cilp*. Correlations were carried out by the Spearman correlation test. Results were considered statistically significant if the *p* < 0.05.

Cardiac fibrosis was evaluated in our model by quantifying the expression of the pro‐fibrotic genes *Tgf*‐β (transforming growth factor‐beta), *Col* I (collagen type I), and α‐*Sma* (alpha‐smooth muscle actin) by qPCR. CILP (cartilage intermediate layer protein‐1), a novel marker of cardiac fibrosis, was also measured. We observed a significant increase in *Tgf*‐β, *Col* I and *Cilp* mRNA levels without any modulation of α‐*Sma* expression in the TAC group compared to the sham group (Figure 3A). Interestingly, *Clu* mRNA levels were positively correlated with those of *Tgf*, *Col I* and *Cilp* (Figure 3B). This correlation is still significantly observed in TAC mice after exclusion of the sham mice group (Figure S2). All these results suggest that CLU may be an indicator of cardiac fibrosis, induced by pressure overload.

### 3.4 Increased Cardiac CLU Levels Are an Indicator of Severe Cardiac Remodelling and Dysfunction

**FIGURE 4:**
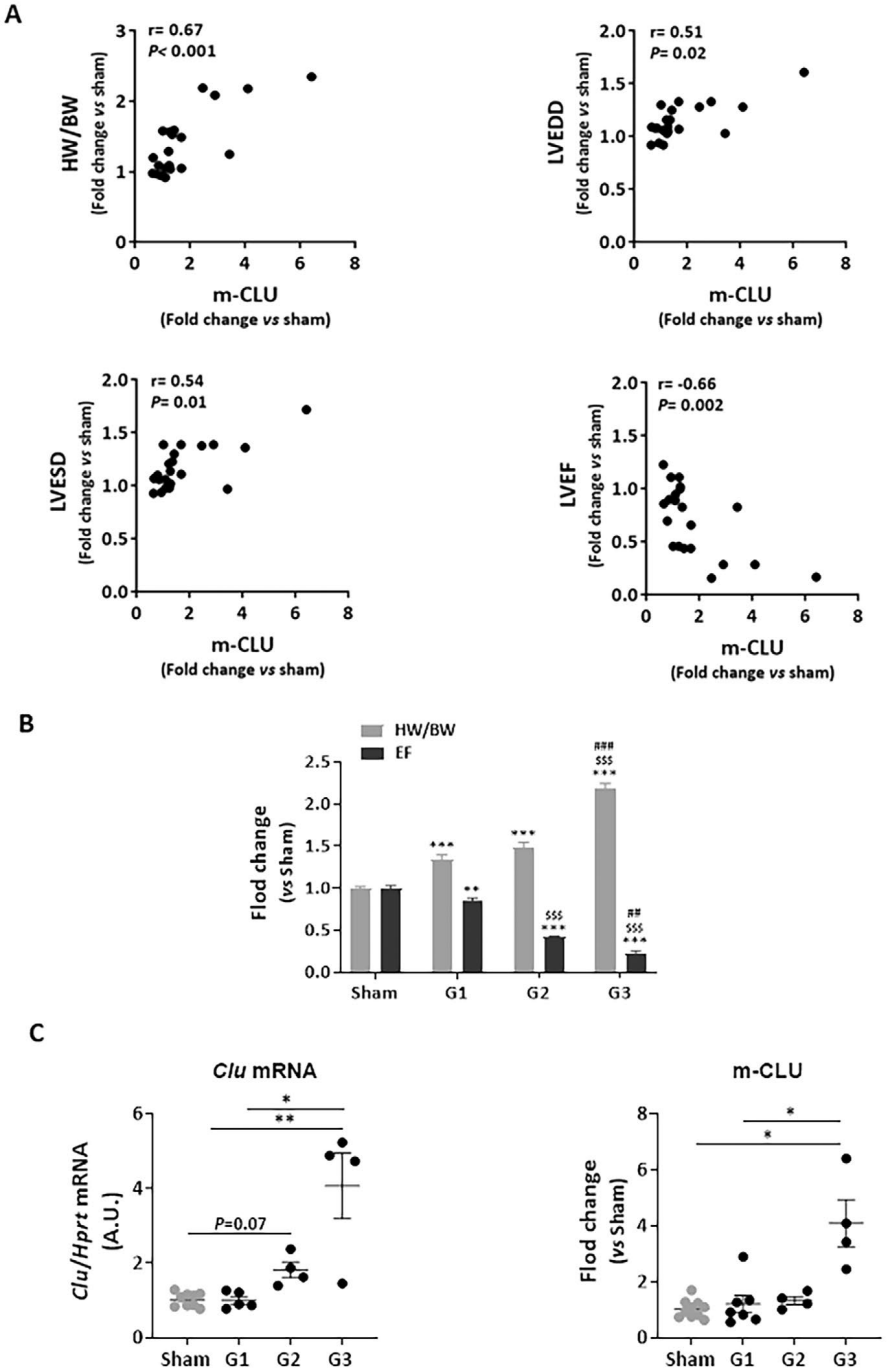
Increased cardiac CLU levels are an indicator of cardiac remodelling and dysfunction induced by transverse aortic constriction. (A) Correlation between intraventricular mature protein levels of CLU (m‐CLU) and HW/BW ratio, LVEDD, LVESD, and EF. Correlations were carried out by the Spearman correlation test. Results were considered statistically significant if the *p* < 0.05. (B) TAC mice were separated into 3 groups depending on severity of cardiac remodelling and dysfunction based on HW/BW ratio and on % of EF, respectively: TAC mice (*n* = 7) with moderate cardiac remodelling and preserved EF (32% increase in HW/BW; EF > 40%); (G2): TAC mice (*n* = 4) with moderate cardiac remodelling and dysfunction (50% increase in HW/BW; EF = 25%); and (G3): TAC mice (*n* = 4) with severe cardiac remodelling and dysfunction (2‐fold increase in HW/BW; EF < 20%). Statistical significance was determined by ANOVA‐ Tukey's multiple comparisons. **p < 0.01, ***p < 0.001 versus sham mice, ^$$$^ *p* < 0.001 versus G1 TAC mice, ^**##**^ *p* < 0.01, ^**###**^ *p* < 0.001 versus G2 TAC mice. (C) Comparison of intraventricular CLU expression (*Clu* mRNA and m‐CLU levels) in sham mice (*n* = 10) and the 3 selected groups of TAC mice. Statistical significance was determined by Kruskal Wallis‐Dunn's multiple comparisons test. *p < 0.05, **p < 0.01.

We first correlated intraventricular CLU levels with heart weight and echocardiographic parameters and found that m‐CLU levels were positively correlated with HW/BW, LVEDD, and LVESD, but negatively correlated with EF (Figure 4A).

We then excluded the TAC mice that did not show cardiac remodelling (mice with a HW/BW ratio similar to the sham mice) and compared the expression of CLU in sham mice and the 3 groups of TAC animals selected based on HW/BW ratio and EF (Figure 4B): G1: TAC mice with moderate cardiac remodelling and preserved EF (40% increase in HW/BW; EF > 40%); G2: TAC mice with moderate cardiac remodelling and altered EF (50% increase in HW/BW; EF = 25%); and G3: TAC mice with severe cardiac remodelling and altered EF (2‐fold increase in HW/BW; EF < 20%). We observed that *Clu* mRNA and m‐CLU levels were significantly increased in TAC mice with severe cardiac remodelling and dysfunction (G3) compared to the sham and TAC G1 mice (Figure 4C).

In parallel, we quantified CLU in the plasma of sham and TAC mice in order to investigate the role of the circulating form of CLU (s‐CLU) as a potential biomarker for TAC‐induced cardiac remodelling and dysfunction. In contrast to *Clu* mRNA and m‐CLU, no significant difference was observed in the TAC mice group compared to the sham mice group as well as after separation of the 3 TAC groups. Furthermore, no correlation was observed between s‐CLU and HW/BW and EF (Figure S3B). These results suggest that CLU expression is induced in the LV of TAC mice during adverse cardiac remodelling. However, plasma CLU levels could not be used as biomarkers of TAC‐induced cardiac remodelling and dysfunction.

To investigate the effect of sex on CLU expression after TAC, we analysed a small number of female mice (4 sham and 4 TAC). As shown in Figure S4A, the phenotype of female TAC mice was similar to the male TAC G1 mice (Figure 4B) based on HW/BW (44% increased) and EF (25% decreased but EF > 40). Interestingly, no difference in CLU mRNA, m‐CLU, or s‐CLU was observed in the TAC female mice compared to the sham (Figure S4B,C), suggesting that CLU regulation during moderate compensated cardiac remodelling after TAC is gender‐independent.

## 4 Discussion

The aim of this study was to investigate whether CLU could be regulated in heart and plasma in response to pressure overload by using a TAC mice model. TAC initially leads to compensated cardiac hypertrophy, which is often associated with a transient improvement in cardiac contractility. Over time, the response to chronic haemodynamic overload becomes maladaptive, leading to cardiac dilatation and eventually HF [18]. We observed for the first time an increase of CLU expression in the LV of the TAC mice, a positive correlation of CLU with cardiac hypertrophy, suggesting that the cardiac levels of CLU could be used as an indicator of excessive hypertrophy induced by pressure overload. This result is consistent with our previous data showing that CLU expression and secretion are increased by isoproterenol‐treated cardiomyocytes and play a pro‐hypertrophic role [15]. To better understand the relationship between CLU expression and cardiac remodelling and dysfunction, we compared the expression of CLU in 3 groups of animals based on the severity of TAC. We showed that CLU is not modulated in LV of mice with moderate TAC (compensated hypertrophy). Our result is consistent with a previous study showing that TAC for only 2 weeks in male mice induced cardiac hypertrophy without HF without increased CLU levels in the LV [12]. We also confirmed this result in female mice, showing that CLU regulation in the heart during compensated hypertrophy after TAC is gender‐independent. Interestingly, we demonstrated for the first time that CLU expression is induced in the LV of TAC mice with severe cardiac remodelling and dysfunction characterised by an excessive alteration of EF with increased LVEDD and LVESD. This result showed that CLU is increased during the maladaptive response leading to dilated cardiomyopathy, a phenotype also observed in response to myocardial infarction. In parallel, it was shown that CLU expression is increased in the right ventricle of animals with severe pulmonary artery constriction [19]. All these results showed that CLU is increased in LV and RV during severe decompensatory hypertrophy in response to pressure overload.

The transition from compensatory to decompensatory hypertrophy is characterised by loss of cardiomyocytes and fibrosis [3, 20]. Several studies showed the protective role of CLU in cardiomyocytes by decreasing apoptosis induced by different stimuli such as oxidative stress and inhibition of protein degradation systems activities [21, 22]. The mechanisms observed during cardiac remodelling and HF post‐injury or pressure overload suggest that CLU may have a protective effect in these models by decreasing the mortality of cardiomyocytes.

Cardiac fibrosis is a pathological process observed during TAC‐induced cardiac remodelling [3, 20]. It is characterised by an excessive deposition of collagen fibres in the heart, leading to myocardial stiffness, impaired cardiac contractility and HF [23]. Several studies showed that CLU could attenuate renal, hepatic and pulmonary fibrosis [24, 25, 26, 27, 28]. However, there was no data on the role of CLU in cardiac fibrosis. In this study we showed a positive correlation between CLU and fibrotic markers suggesting that CLU may be an indicator of cardiac fibrosis, induced by pressure overload. This result is not necessarily in contradiction with previous studies showing an anti‐fibrotic effect of CLU. Indeed, we observed that CLU is positively correlated with CILP, a novel marker of cardiac fibrosis that is upregulated in TAC mice and plays an anti‐fibrotic role [29, 30, 31]. As CILP [30, 32], CLU may be secreted by hypertrophied cardiomyocytes and/or activated fibroblasts for inhibiting cardiac fibrosis. Further studies are needed to investigate the role of CLU in this particular process.

Conversely, in the case of intraventricular CLU levels, no change in plasma CLU levels was observed in all groups of TAC mice, showing that plasma CLU levels could not be used as biomarkers of TAC‐induced cardiac remodelling and dysfunction. It should be noted that CLU is ubiquitously expressed, and its plasma level reflects its secretion by all organs, with the liver accounting for the majority [33]. In our previous study, we have shown that CLU expression is induced in the heart of MI‐ rats without any difference in their plasma CLU levels [15]. However, plasma CLU levels are increased and positively correlated with cardiac remodelling in patients after MI. Further studies are needed to investigate the role of the circulating form of CLU in cardiac remodelling and dysfunction induced by pressure overload.

In conclusion, we have shown for the first time that CLU expression is induced in the LV due to chronic pressure overload in mice and that its level can be used as an indicator of severe cardiac remodelling and contractile dysfunction. As CLU is a ubiquitous protein, it will be important to investigate if CLU is regulated in cardiomyocytes and/or cardiac fibroblasts under stretch conditions to better understand the mechanisms involved in its regulation and whether CLU protects against cardiac hypertrophy and fibrosis or whether it is involved in these processes.

## Author Contributions

**Annie Turkieh:** conceptualization (equal), data curation (lead), formal analysis (equal), writing – original draft (equal). **Lukas Weber:** methodology (equal), resources (equal). **Maggy Chwastyniak:** investigation (equal), methodology (equal). **Simge Baydar:** methodology (equal), resources (equal). **Olivia Beseme:** methodology, investigation. **Matthias Ernst:** investigation. **Quianling Ye:** investigation. **Philippe Amouyel:** formal analysis (equal), resources (equal). **Bruno K. Podesser:** resources (equal). **Attila Kiss:** resources (equal), supervision (equal), writing – review and editing (equal). **Florence Pinet:** conceptualization (equal), formal analysis (equal), funding acquisition (equal), resources (equal), supervision (equal), writing – original draft (equal).

## Conflicts of Interest

The authors declare no conflicts of interest.

## Supporting information


Appendix S1.

## Data Availability

The data that support the findings of this study are available from the corresponding author upon reasonable request.
