# Supplementary material for: Regulation of Clusterin in the Heart and Plasma of Mice After Transverse Aortic Constriction
Source: J Cell Mol Med. 2024 Dec 13;28(23):e70290. doi: 10.1111/jcmm.70290 (PMC11640023; doi:10.1111/jcmm.70290)
Supplement: Supplementary file 1 — Appendix S1. [file JCMM-28-e70290-s001.docx]

**Supplemental data**

#### Regulation of Clusterin in the heart and plasma of mice after transverse aortic constriction

#### Annie Turkieh^1*^, Lukas Weber^2^, Maggy Chwastyniak^1^, Simge Baydar^2^, Olivia Beseme^1^, Matthias Ernst^2^, Quian-Ling Ye^2^, Philippe Amouyel^1^, Bruno K Podesser ^2^, Attila Kiss^2^, Florence Pinet^1*^

^1^ Université de Lille, Inserm, CHU Lille, Institut Pasteur de Lille, U1167- RID-AGE, Lille, France

^2^ Ludwig Boltzmann Institute for Cardiovascular Research at the Center for Biomedical Research and Translational Surgery, Medical University of Vienna, Vienna, Austria

***** Corresponding authors: [annie.turkieh@pasteur-lille.fr](mailto:annie.turkieh@pasteur-lille.fr); +33 (0)3 20 87 73 62; [florence.pinet@pasteur-lille.fr](mailto:florence.pinet@pasteur-lille.fr); +33 (0)3 20 87 73 72

**Table S1: The sequences and the melting temperature of the different primers used for qPCR analysis**

| Gene | Primers sequences | Melting temperature |
| --- | --- | --- |
| *Anp* | F : TATTGGAGCAAATCCTGTGT  R : TAGCAGGTTCTTGAAATCCA | 56 |
| *Bnp* | F : AAGTCCTAGCCAGTCTCCAG  R : TCCTTCAAGAGCTGTCTCTG | 56 |
| *α-Mhc* | F : ATTTCTCCAACCCAGGATCT  R : ACAGGCAGGAAGAGGAGTAG | 56 |
| *β-Mhc* | F : CCTACGATTATGCGTTCATC  R : CCTGTCAGCTTGTAAATGGA | 56 |
| *α-Sma* | F : GTCCCAGACATCAGGGAGTAA  R : TCGGATACTTCAGCGTCAGGA | 49 |
| *Col-I* | F : GTGTTCCCTACTCAGCCGTC  R : ACTCGAACGGGAATCCATCG | 62 |
| *Tgf-β* | F : CTCACCGCGACTCCTGCTGC  R : TCGGAGAGCGGGAACCCTCG | 52 |
| *Cilp* | F : CCAGGAGAGTCCCGGAGAG  R : CATCCAGCCGCTCATAGTCA | 54 |
| *Clu* | F : GATGATCCACCAGGCTCAACAG  R : ACACAGTGCGGTCATCTTCACC | 54 |
| *Hprt*  *18s* | F : AAAGGACCTCTCGAAGTGTT  R : TGACACAAACGTGATTCAAA  F: TCGTATTGCGCCGCTAGAG  R: TGAAAACATTCTTGGCAAATGC | 52  55 |

**
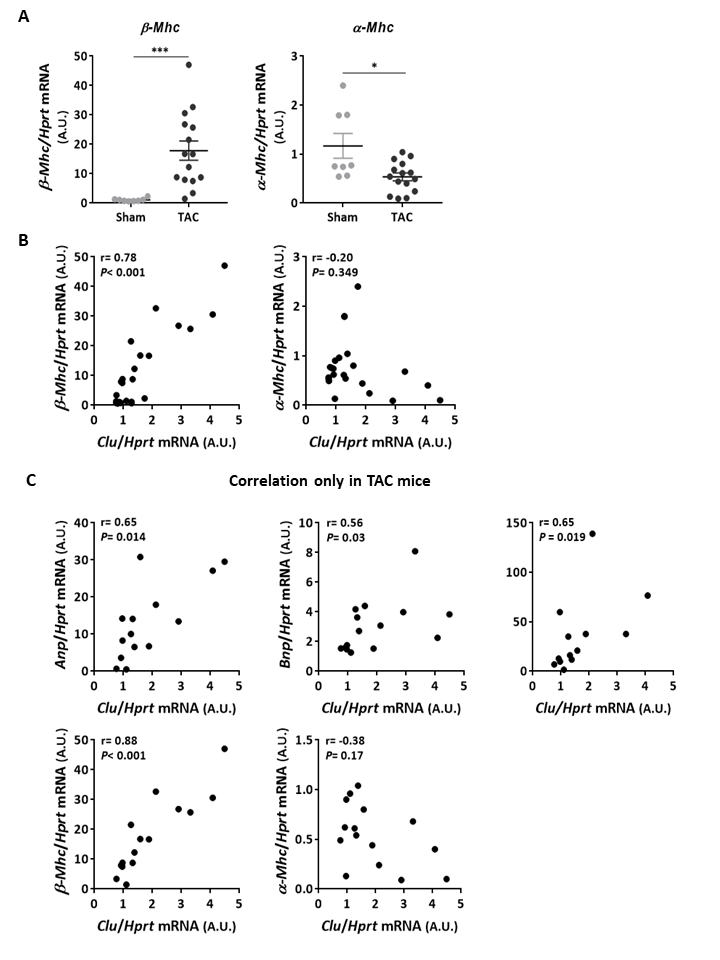
**

**Figure S1: CLU level is positively correlated to cardiac hypertrophy in TAC mice. A.** Quantification by qPCR of mRNA levels of α- and β-*Mhc*. *Hprt* was used for normalization. Statistical significance was determined by Wilcoxon-Mann Whitney test * *p* < 0.05, ** *p* < 0.01, and *** *p* < 0.001. **B.** Correlation between intraventricular *Clu* mRNA levels and α- and β-*Mhc*. **C**. Correlation between intraventricular *Clu* mRNA levels and of *Anp*, *Bnp*, α- and β-*Mhc* after sham exclusion. **D.** Correlation between intraventricular CLU mature form (m-CLU) and HW/BW ratio. Correlations were carried out by Spearman correlation test. Results were considered statistically significant if P< 0.05.

**
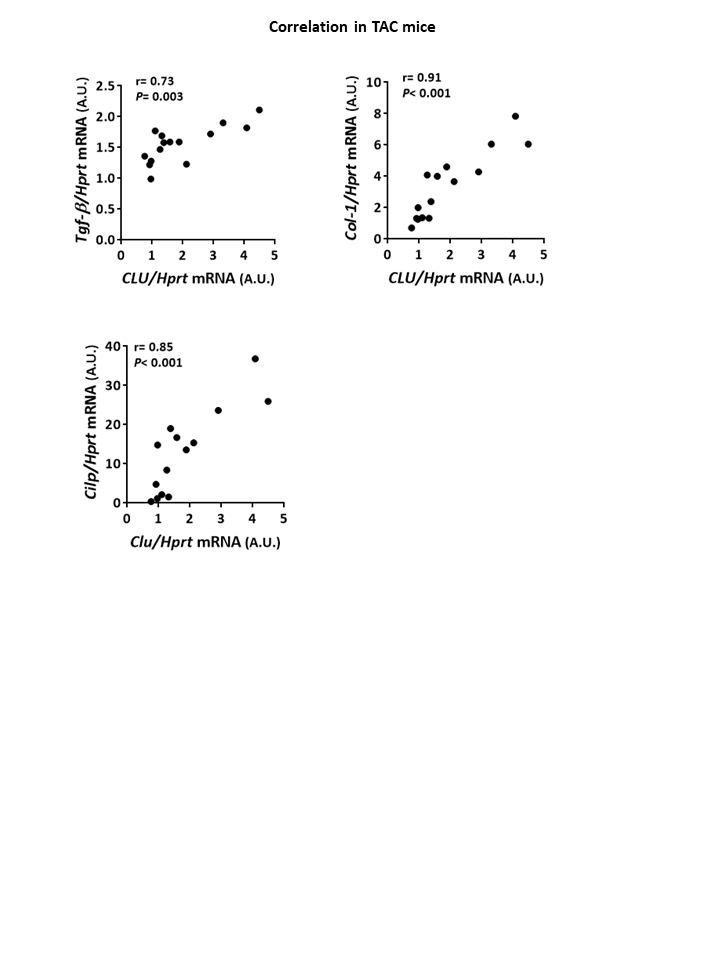
**

**Figure S2: CLU level is positively correlated to cardiac fibrosis in TAC mice.** Correlation between intraventricular *Clu* mRNA levels and *Tgf-β*, *Col I* and *Cilp* after sham exclusion. Correlations were carried out by Spearman correlation test. Results were considered statistically significant if P < 0.05.

**
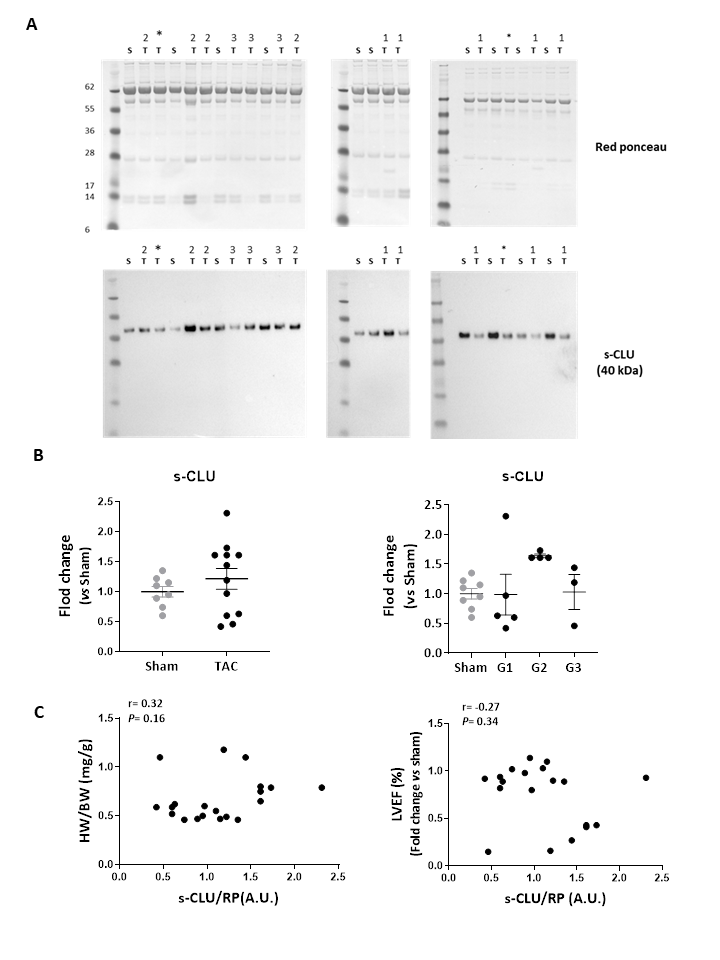
**

**Figure S3: Plasma CLU levels are not correlated to cardiac remodeling and dysfunction induced by transverse aortic constriction. A.** Red ponceau staining and western blot of CLU levels in plasma of sham and TAC mice. S: sham, T: TAC, 1: G1, 2: G2, 3: G3. * indicate the samples unused in this study. **B.** Quantification of plasma CLU (s-CLU) levels in sham and all TAC groups together (left panel) or the 3 groups TAC separately (right panel) **C;** Correlation between s-CLU and HW/BW ratio (left panel) and between s-CLU and EF (left panel). Correlations were carried out by Spearman correlation test. Results were considered statistically significant if P < 0.05.

**
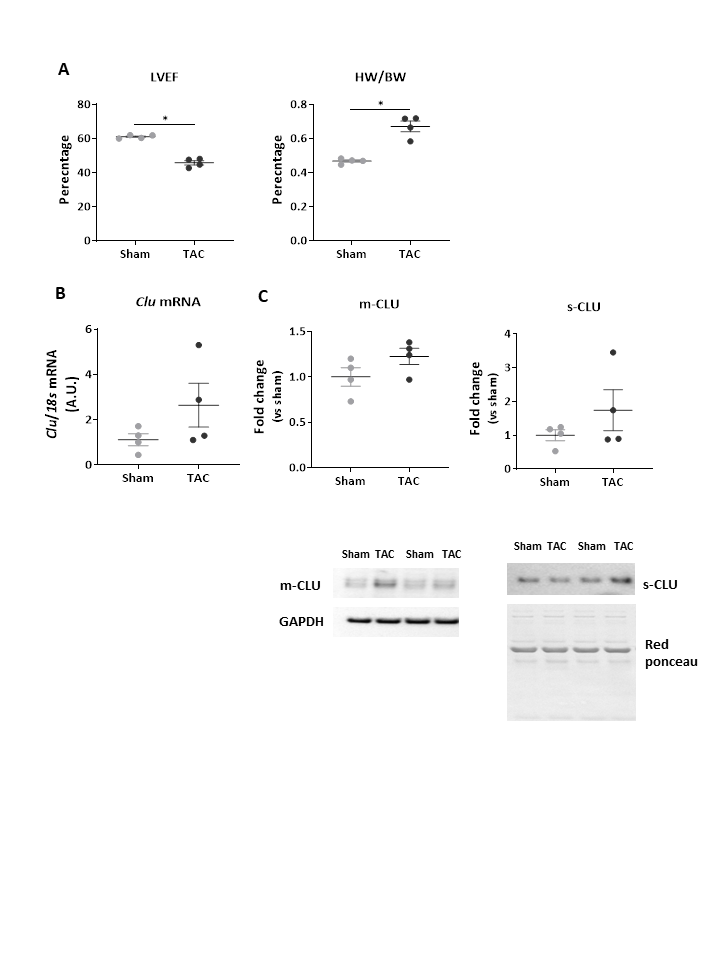
**

**Figure S4: CLU regulation in heart and plasma of female mice. A.** HW/BW ratio and EF of sham (n=4) and TAC (n=4) mice. **B.** Quantification of *Clu* mRNA levels by qPCR in left ventricle of sham and TAC mice. *18s* was used for normalization. **C.** Representative image and quantification by western blot of mature protein form in left ventricle (m-CLU) and plasma (s-CLU) in the same samples. GAPDH and red ponceau were used to normalize m-CLU and s-CLU, respectively.
